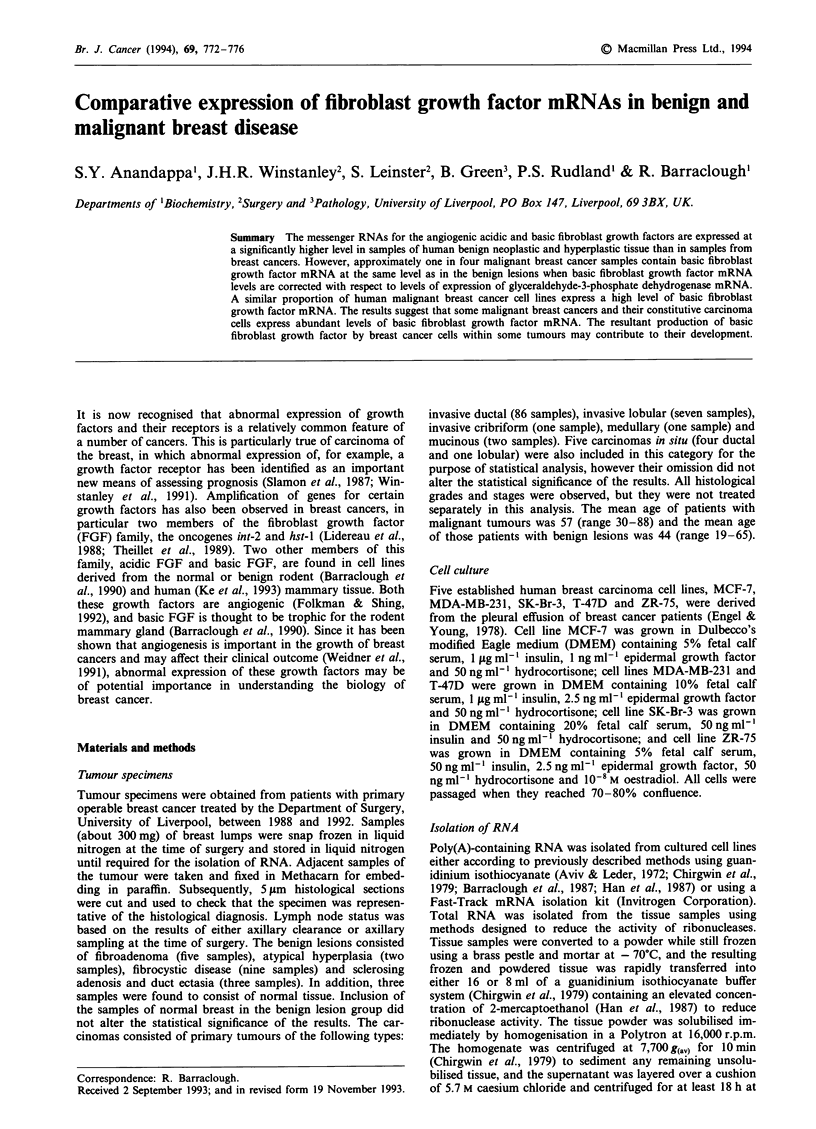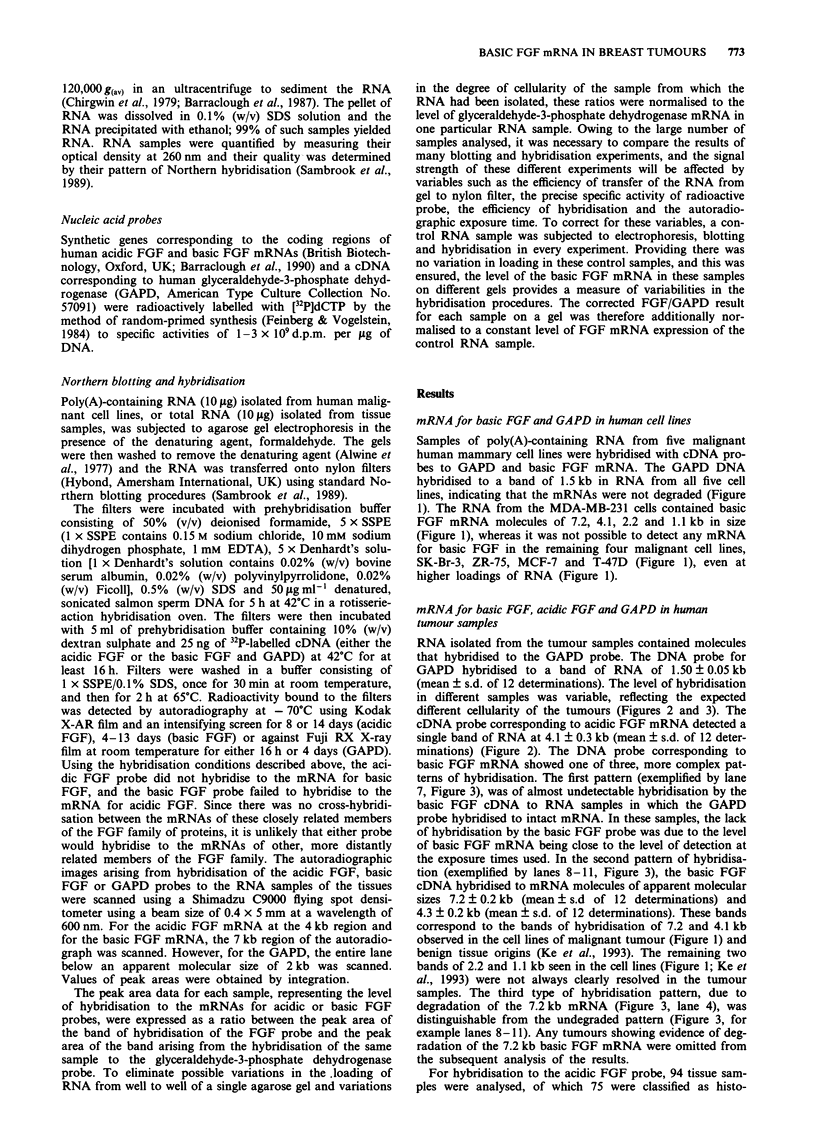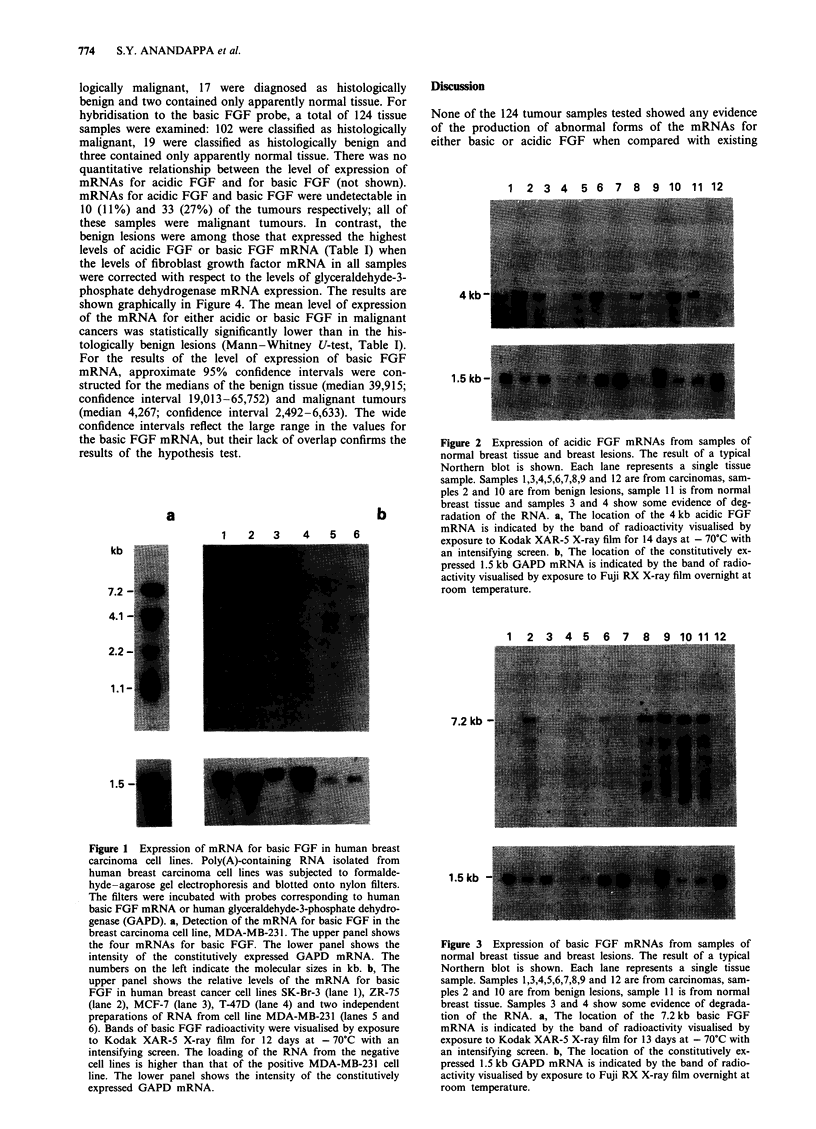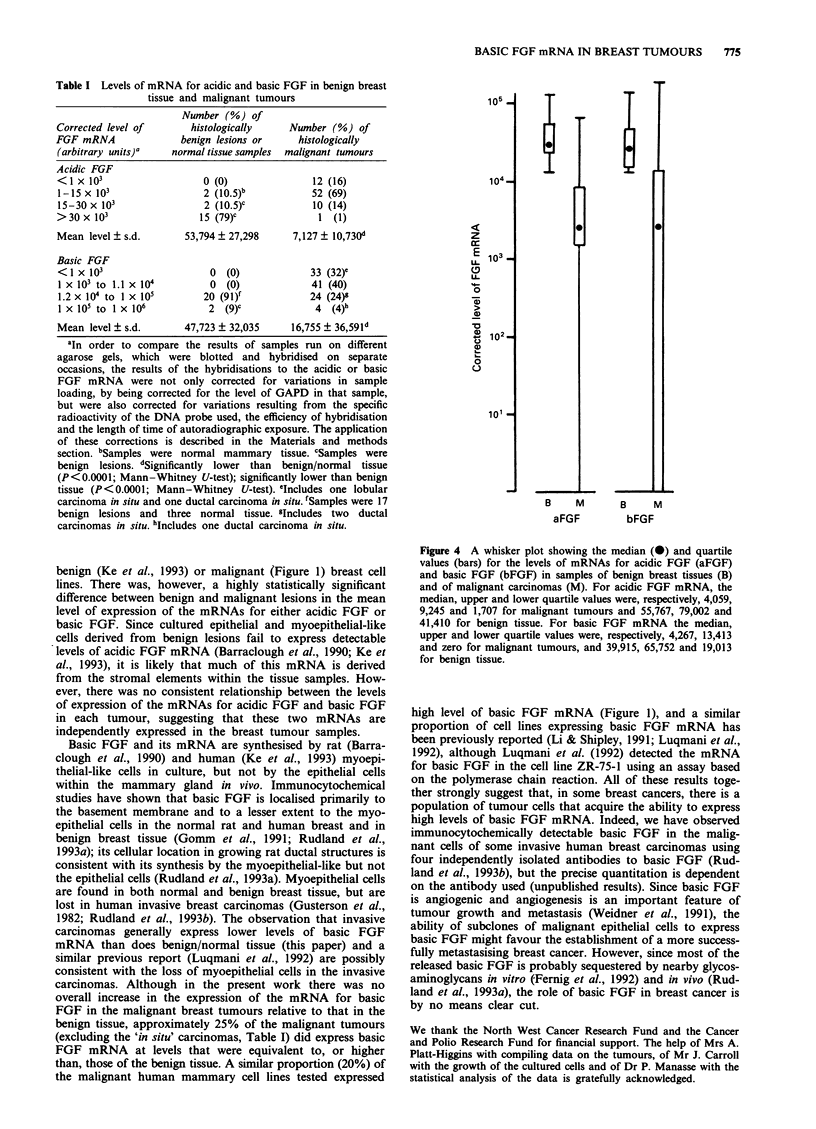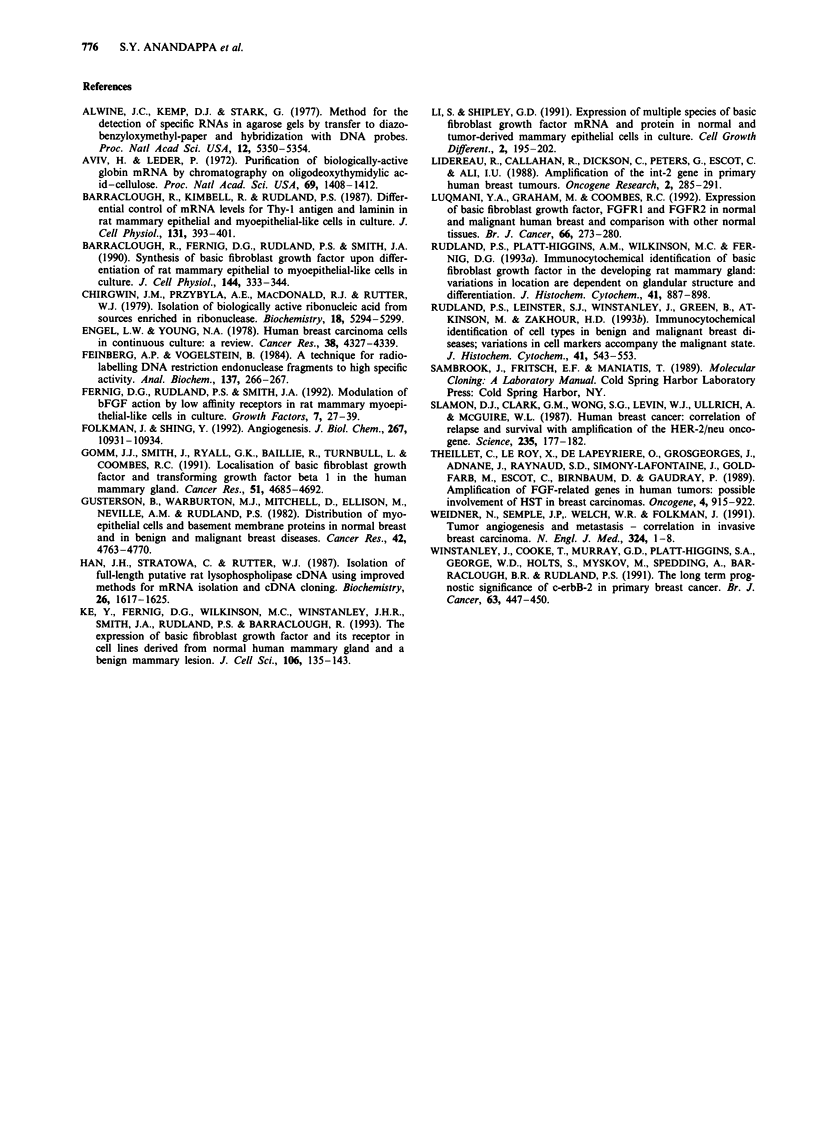# Comparative expression of fibroblast growth factor mRNAs in benign and malignant breast disease.

**DOI:** 10.1038/bjc.1994.146

**Published:** 1994-04

**Authors:** S. Y. Anandappa, J. H. Winstanley, S. Leinster, B. Green, P. S. Rudland, R. Barraclough

**Affiliations:** Department of Biochemistry, University of Liverpool, UK.

## Abstract

**Images:**


Br. J. Cancer (1994), 69, 772-776 © Macmillan Press Ltd., 1994

### Comparative expression of fibroblast growth factor mRNAs in benign and malignant breast disease

S.Y. Anandappa[1], J.H.R. Winstanley[2], S. Leinster[2], B. Green[3], P.S. Rudland[1] & R. Barraclough[1]

*Departments of [1]Biochemistry, [2]Surgery and [3]Pathology, University of Liverpool, PO Box 147, Liverpool, 69 3BX, UK.*

**Summary** The messenger RNAs for the angiogenic acidic and basic fibroblast growth factors are expressed at a significantly higher level in samples of human benign neoplastic and hyperplastic tissue than in samples from breast cancers. However, approximately one in four malignant breast cancer samples contain basic fibroblast growth factor mRNA at the same level as in the benign lesions when basic fibroblast growth factor mRNA levels are corrected with respect to levels of expression of glyceraldehyde-3-phosphate dehydrogenase mRNA. A similar proportion of human malignant breast cancer cell lines express a high level of basic fibroblast growth factor mRNA. The results suggest that some malignant breast cancers and their constitutive carcinoma cells express abundant levels of basic fibroblast growth factor mRNA. The resultant production of basic fibroblast growth factor by breast cancer cells within some tumours may contribute to their development.

It is now recognised that abnormal expression of growth factors and their receptors is a relatively common feature of a number of cancers. This is particularly true of carcinoma of the breast, in which abnormal expression of, for example, a growth factor receptor has been identified as an important new means of assessing prognosis (Slamon *et al.*, 1987; Winstanley *et al.*, 1991). Amplification of genes for certain growth factors has also been observed in breast cancers, in particular two members of the fibroblast growth factor (FGF) family, the oncogenes *int*-2 and *hst*-1 (Lidereau *et al.*, 1988; Theillet *et al.*, 1989). Two other members of this family, acidic FGF and basic FGF, are found in cell lines derived from the normal or benign rodent (Barraclough *et al.*, 1990) and human (Ke *et al.*, 1993) mammary tissue. Both these growth factors are angiogenic (Folkman & Shing, 1992), and basic FGF is thought to be trophic for the rodent mammary gland (Barraclough *et al.*, 1990). Since it has been shown that angiogenesis is important in the growth of breast cancers and may affect their clinical outcome (Weidner *et al.*, 1991), abnormal expression of these growth factors may be of potential importance in understanding the biology of breast cancer.

### Materials and methods

### *Tumour specimens*

Tumour specimens were obtained from patients with primary operable breast cancer treated by the Department of Surgery, University of Liverpool, between 1988 and 1992. Samples (about 300 mg) of breast lumps were snap frozen in liquid nitrogen at the time of surgery and stored in liquid nitrogen until required for the isolation of RNA. Adjacent samples of the tumour were taken and fixed in Methacarn for embedding in paraffin. Subsequently, 5 $\mu$m histological sections were cut and used to check that the specimen was representative of the histological diagnosis. Lymph node status was based on the results of either axillary clearance or axillary sampling at the time of surgery. The benign lesions consisted of fibroadenoma (five samples), atypical hyperplasia (two samples), fibrocystic disease (nine samples) and sclerosing adenosis and duct ectasia (three samples). In addition, three samples were found to consist of normal tissue. Inclusion of the samples of normal breast in the benign lesion group did not alter the statistical significance of the results. The carcinomas consisted of primary tumours of the following types: invasive ductal (86 samples), invasive lobular (seven samples), invasive cribriform (one sample), medullary (one sample) and mucinous (two samples). Five carcinomas *in situ* (four ductal and one lobular) were also included in this category for the purpose of statistical analysis, however their omission did not alter the statistical significance of the results. All histological grades and stages were observed, but they were not treated separately in this analysis. The mean age of patients with malignant tumours was 57 (range 30-88) and the mean age of those patients with benign lesions was 44 (range 19-65).

### *Cell culture*

Five established human breast carcinoma cell lines, MCF-7, MDA-MB-231, SK-Br-3, T-47D and ZR-75, were derived from the pleural effusion of breast cancer patients (Engel & Young, 1978). Cell line MCF-7 was grown in Dulbecco's modified Eagle medium (DMEM) containing 5% fetal calf serum, 1 $\mu g\ ml^{-1}$ insulin, 1 $ng\ ml^{-1}$ epidermal growth factor and 50 $ng\ ml^{-1}$ hydrocortisone; cell lines MDA-MB-231 and T-47D were grown in DMEM containing 10% fetal calf serum, 1 $\mu g\ ml^{-1}$ insulin, 2.5 $ng\ ml^{-1}$ epidermal growth factor and 50 $ng\ ml^{-1}$ hydrocortisone; cell line SK-Br-3 was grown in DMEM containing 20% fetal calf serum, 50 $ng\ ml^{-1}$ insulin and 50 $ng\ ml^{-1}$ hydrocortisone; and cell line ZR-75 was grown in DMEM containing 5% fetal calf serum, 50 $ng\ ml^{-1}$ insulin, 2.5 $ng\ ml^{-1}$ epidermal growth factor, 50 $ng\ ml^{-1}$ hydrocortisone and $10^{-8}$ M oestradiol. All cells were passaged when they reached 70-80% confluence.

### *Isolation of RNA*

Poly(A)-containing RNA was isolated from cultured cell lines either according to previously described methods using guanidinium isothiocyanate (Aviv & Leder, 1972; Chirgwin *et al.*, 1979; Barraclough *et al.*, 1987; Han *et al.*, 1987) or using a Fast-Track mRNA isolation kit (Invitrogen Corporation). Total RNA was isolated from the tissue samples using methods designed to reduce the activity of ribonucleases. Tissue samples were converted to a powder while still frozen using a brass pestle and mortar at − 70°C, and the resulting frozen and powdered tissue was rapidly transferred into either 16 or 8 ml of a guanidinium isothiocyanate buffer system (Chirgwin *et al.*, 1979) containing an elevated concentration of 2-mercaptoethanol (Han *et al.*, 1987) to reduce ribonuclease activity. The tissue powder was solubilised immediately by homogenisation in a Polytron at 16,000 r.p.m. The homogenate was centrifuged at 7,700 $g_{(av)}$ for 10 min (Chirgwin *et al.*, 1979) to sediment any remaining unsolubilised tissue, and the supernatant was layered over a cushion of 5.7 M caesium chloride and centrifuged for at least 18 h at

Correspondence: R. Barraclough.
Received 2 September 1993; and in revised form 19 November 1993.

120,000 $g_{(av)}$ in an ultracentrifuge to sediment the RNA (Chirgwin *et al.*, 1979; Barraclough *et al.*, 1987). The pellet of RNA was dissolved in 0.1% (w/v) SDS solution and the RNA precipitated with ethanol; 99% of such samples yielded RNA. RNA samples were quantified by measuring their optical density at 260 nm and their quality was determined by their pattern of Northern hybridisation (Sambrook *et al.*, 1989).

*Nucleic acid probes*

Synthetic genes corresponding to the coding regions of human acidic FGF and basic FGF mRNAs (British Biotechnology, Oxford, UK; Barraclough *et al.*, 1990) and a cDNA corresponding to human glyceraldehyde-3-phosphate dehydrogenase (GAPD, American Type Culture Collection No. 57091) were radioactively labelled with [$^{32}$P]dCTP by the method of random-primed synthesis (Feinberg & Vogelstein, 1984) to specific activities of $1-3 \times 10^9$ d.p.m. per μg of DNA.

*Northern blotting and hybridisation*

Poly(A)-containing RNA (10 μg) isolated from human malignant cell lines, or total RNA (10 μg) isolated from tissue samples, was subjected to agarose gel electrophoresis in the presence of the denaturing agent, formaldehyde. The gels were then washed to remove the denaturing agent (Alwine *et al.*, 1977) and the RNA was transferred onto nylon filters (Hybond, Amersham International, UK) using standard Northern blotting procedures (Sambrook *et al.*, 1989).

The filters were incubated with prehybridisation buffer consisting of 50% (v/v) deionised formamide, 5 × SSPE (1 × SSPE contains 0.15 M sodium chloride, 10 mM sodium dihydrogen phosphate, 1 mM EDTA), 5 × Denhardt's solution [1 × Denhardt's solution contains 0.02% (w/v) bovine serum albumin, 0.02% (w/v) polyvinylpyrrolidone, 0.02% (w/v) Ficoll], 0.5% (w/v) SDS and 50 μg ml$^{-1}$ denatured, sonicated salmon sperm DNA for 5 h at 42°C in a rotisserie-action hybridisation oven. The filters were then incubated with 5 ml of prehybridisation buffer containing 10% (w/v) dextran sulphate and 25 ng of $^{32}$P-labelled cDNA (either the acidic FGF or the basic FGF and GAPD) at 42°C for at least 16 h. Filters were washed in a buffer consisting of 1 × SSPE/0.1% SDS, once for 30 min at room temperature, and then for 2 h at 65°C. Radioactivity bound to the filters was detected by autoradiography at − 70°C using Kodak X-AR film and an intensifying screen for 8 or 14 days (acidic FGF), 4–13 days (basic FGF) or against Fuji RX X-ray film at room temperature for either 16 h or 4 days (GAPD). Using the hybridisation conditions described above, the acidic FGF probe did not hybridise to the mRNA for basic FGF, and the basic FGF probe failed to hybridise to the mRNA for acidic FGF. Since there was no cross-hybridisation between the mRNAs of these closely related members of the FGF family of proteins, it is unlikely that either probe would hybridise to the mRNAs of other, more distantly related members of the FGF family. The autoradiographic images arising from hybridisation of the acidic FGF, basic FGF or GAPD probes to the RNA samples of the tissues were scanned using a Shimadzu C9000 flying spot densitometer using a beam size of 0.4 × 5 mm at a wavelength of 600 nm. For the acidic FGF mRNA at the 4 kb region and for the basic FGF mRNA, the 7 kb region of the autoradiograph was scanned. However, for the GAPD, the entire lane below an apparent molecular size of 2 kb was scanned. Values of peak areas were obtained by integration.

The peak area data for each sample, representing the level of hybridisation to the mRNAs for acidic or basic FGF probes, were expressed as a ratio between the peak area of the band of hybridisation of the FGF probe and the peak area of the band arising from the hybridisation of the same sample to the glyceraldehyde-3-phosphate dehydrogenase probe. To eliminate possible variations in the .loading of RNA from well to well of a single agarose gel and variations in the degree of cellularity of the sample from which the RNA had been isolated, these ratios were normalised to the level of glyceraldehyde-3-phosphate dehydrogenase mRNA in one particular RNA sample. Owing to the large number of samples analysed, it was necessary to compare the results of many blotting and hybridisation experiments, and the signal strength of these different experiments will be affected by variables such as the efficiency of transfer of the RNA from gel to nylon filter, the precise specific activity of radioactive probe, the efficiency of hybridisation and the autoradiographic exposure time. To correct for these variables, a control RNA sample was subjected to electrophoresis, blotting and hybridisation in every experiment. Providing there was no variation in loading in these control samples, and this was ensured, the level of the basic FGF mRNA in these samples on different gels provides a measure of variabilities in the hybridisation procedures. The corrected FGF/GAPD result for each sample on a gel was therefore additionally normalised to a constant level of FGF mRNA expression of the control RNA sample.

### Results

*mRNA for basic FGF and GAPD in human cell lines*

Samples of poly(A)-containing RNA from five malignant human mammary cell lines were hybridised with cDNA probes to GAPD and basic FGF mRNA. The GAPD DNA hybridised to a band of 1.5 kb in RNA from all five cell lines, indicating that the mRNAs were not degraded (Figure 1). The RNA from the MDA-MB-231 cells contained basic FGF mRNA molecules of 7.2, 4.1, 2.2 and 1.1 kb in size (Figure 1), whereas it was not possible to detect any mRNA for basic FGF in the remaining four malignant cell lines, SK-Br-3, ZR-75, MCF-7 and T-47D (Figure 1), even at higher loadings of RNA (Figure 1).

*mRNA for basic FGF, acidic FGF and GAPD in human tumour samples*

RNA isolated from the tumour samples contained molecules that hybridised to the GAPD probe. The DNA probe for GAPD hybridised to a band of RNA of 1.50 ± 0.05 kb (mean ± s.d. of 12 determinations). The level of hybridisation in different samples was variable, reflecting the expected different cellularity of the tumours (Figures 2 and 3). The cDNA probe corresponding to acidic FGF mRNA detected a single band of RNA at 4.1 ± 0.3 kb (mean ± s.d. of 12 determinations) (Figure 2). The DNA probe corresponding to basic FGF mRNA showed one of three, more complex patterns of hybridisation. The first pattern (exemplified by lane 7, Figure 3), was of almost undetectable hybridisation by the basic FGF cDNA to RNA samples in which the GAPD probe hybridised to intact mRNA. In these samples, the lack of hybridisation by the basic FGF probe was due to the level of basic FGF mRNA being close to the level of detection at the exposure times used. In the second pattern of hybridisation (exemplified by lanes 8–11, Figure 3), the basic FGF cDNA hybridised to mRNA molecules of apparent molecular sizes 7.2 ± 0.2 kb (mean ± s.d of 12 determinations) and 4.3 ± 0.2 kb (mean ± s.d. of 12 determinations). These bands correspond to the bands of hybridisation of 7.2 and 4.1 kb observed in the cell lines of malignant tumour (Figure 1) and benign tissue origins (Ke *et al.*, 1993). The remaining two bands of 2.2 and 1.1 kb seen in the cell lines (Figure 1; Ke *et al.*, 1993) were not always clearly resolved in the tumour samples. The third type of hybridisation pattern, due to degradation of the 7.2 kb mRNA (Figure 3, lane 4), was distinguishable from the undegraded pattern (Figure 3, for example lanes 8–11). Any tumours showing evidence of degradation of the 7.2 kb basic FGF mRNA were omitted from the subsequent analysis of the results.

For hybridisation to the acidic FGF probe, 94 tissue samples were analysed, of which 75 were classified as histologically malignant, 17 were diagnosed as histologically benign and two contained only apparently normal tissue. For hybridisation to the basic FGF probe, a total of 124 tissue samples were examined: 102 were classified as histologically malignant, 19 were classified as histologically benign and three contained only apparently normal tissue. There was no quantitative relationship between the level of expression of mRNAs for acidic FGF and for basic FGF (not shown). mRNAs for acidic FGF and basic FGF were undetectable in 10 (11%) and 33 (27%) of the tumours respectively; all of these samples were malignant tumours. In contrast, the benign lesions were among those that expressed the highest levels of acidic FGF or basic FGF mRNA (Table I) when the levels of fibroblast growth factor mRNA in all samples were corrected with respect to the levels of glyceraldehyde-3-phosphate dehydrogenase mRNA expression. The results are shown graphically in Figure 4. The mean level of expression of the mRNA for either acidic or basic FGF in malignant cancers was statistically significantly lower than in the histologically benign lesions (Mann–Whitney *U*-test, Table I). For the results of the level of expression of basic FGF mRNA, approximate 95% confidence intervals were constructed for the medians of the benign tissue (median 39,915; confidence interval 19,013–65,752) and malignant tumours (median 4,267; confidence interval 2,492–6,633). The wide confidence intervals reflect the large range in the values for the basic FGF mRNA, but their lack of overlap confirms the results of the hypothesis test.

**Figure 1** Expression of mRNA for basic FGF in human breast carcinoma cell lines. Poly(A)-containing RNA isolated from human breast carcinoma cell lines was subjected to formaldehyde–agarose gel electrophoresis and blotted onto nylon filters. The filters were incubated with probes corresponding to human basic FGF mRNA or human glyceraldehyde-3-phosphate dehydrogenase (GAPD). **a**, Detection of the mRNA for basic FGF in the breast carcinoma cell line, MDA-MB-231. The upper panel shows the four mRNAs for basic FGF. The lower panel shows the intensity of the constitutively expressed GAPD mRNA. The numbers on the left indicate the molecular sizes in kb. **b**, The upper panel shows the relative levels of the mRNA for basic FGF in human breast cancer cell lines SK-Br-3 (lane 1), ZR-75 (lane 2), MCF-7 (lane 3), T-47D (lane 4) and two independent preparations of RNA from cell line MDA-MB-231 (lanes 5 and 6). Bands of basic FGF radioactivity were visualised by exposure to Kodak XAR-5 X-ray film for 12 days at − 70°C with an intensifying screen. The loading of the RNA from the negative cell lines is higher than that of the positive MDA-MB-231 cell line. The lower panel shows the intensity of the constitutively expressed GAPD mRNA.

### Discussion

None of the 124 tumour samples tested showed any evidence of the production of abnormal forms of the mRNAs for either basic or acidic FGF when compared with existing

**Figure 2** Expression of acidic FGF mRNAs from samples of normal breast tissue and breast lesions. The result of a typical Northern blot is shown. Each lane represents a single tissue sample. Samples 1,3,4,5,6,7,8,9 and 12 are from carcinomas, samples 2 and 10 are from benign lesions, sample 11 is from normal breast tissue and samples 3 and 4 show some evidence of degradation of the RNA. **a**, The location of the 4 kb acidic FGF mRNA is indicated by the band of radioactivity visualised by exposure to Kodak XAR-5 X-ray film for 14 days at − 70°C with an intensifying screen. **b**, The location of the constitutively expressed 1.5 kb GAPD mRNA is indicated by the band of radioactivity visualised by exposure to Fuji RX X-ray film overnight at room temperature.

**Figure 3** Expression of basic FGF mRNAs from samples of normal breast tissue and breast lesions. The result of a typical Northern blot is shown. Each lane represents a single tissue sample. Samples 1,3,4,5,6,7,8,9 and 12 are from carcinomas, samples 2 and 10 are from benign lesions, sample 11 is from normal breast tissue. Samples 3 and 4 show some evidence of degradation of the RNA. **a**, The location of the 7.2 kb basic FGF mRNA is indicated by the band of radioactivity visualised by exposure to Kodak XAR-5 X-ray film for 13 days at − 70°C with an intensifying screen. **b**, The location of the constitutively expressed 1.5 kb GAPD mRNA is indicated by the band of radioactivity visualised by exposure to Fuji RX X-ray film overnight at room temperature.

**Table I** Levels of mRNA for acidic and basic FGF in benign breast tissue and malignant tumours

| *Corrected level of FGF mRNA (arbitrary units)*[a] | *Number (%) of histologically benign lesions or normal tissue samples* | *Number (%) of histologically malignant tumours* |
|---|---|---|
| *Acidic FGF* | | |
| $<1 \times 10^3$ | 0 (0) | 12 (16) |
| $1-15 \times 10^3$ | 2 (10.5)[b] | 52 (69) |
| $15-30 \times 10^3$ | 2 (10.5)[c] | 10 (14) |
| $>30 \times 10^3$ | 15 (79)[c] | 1 (1) |
| Mean level ± s.d. | 53,794 ± 27,298 | 7,127 ± 10,730[d] |
| *Basic FGF* | | |
| $<1 \times 10^3$ | 0 (0) | 33 (32)[e] |
| $1 \times 10^3$ to $1.1 \times 10^4$ | 0 (0) | 41 (40) |
| $1.2 \times 10^4$ to $1 \times 10^5$ | 20 (91)[f] | 24 (24)[g] |
| $1 \times 10^5$ to $1 \times 10^6$ | 2 (9)[c] | 4 (4)[h] |
| Mean level ± s.d. | 47,723 ± 32,035 | 16,755 ± 36,591[d] |

[a]In order to compare the results of samples run on different agarose gels, which were blotted and hybridised on separate occasions, the results of the hybridisations to the acidic or basic FGF mRNA were not only corrected for variations in sample loading, by being corrected for the level of GAPD in that sample, but were also corrected for variations resulting from the specific radioactivity of the DNA probe used, the efficiency of hybridisation and the length of time of autoradiographic exposure. The application of these corrections is described in the Materials and methods section. [b]Samples were normal mammary tissue. [c]Samples were benign lesions. [d]Significantly lower than benign/normal tissue ($P<0.0001$; Mann–Whitney *U*-test); significantly lower than benign tissue ($P<0.0001$; Mann–Whitney *U*-test). [e]Includes one lobular carcinoma *in situ* and one ductal carcinoma *in situ*. [f]Samples were 17 benign lesions and three normal tissue. [g]Includes two ductal carcinomas *in situ*. [h]Includes one ductal carcinoma *in situ*.

**Figure 4** A whisker plot showing the median (●) and quartile values (bars) for the levels of mRNAs for acidic FGF (aFGF) and basic FGF (bFGF) in samples of benign breast tissues (B) and of malignant carcinomas (M). For acidic FGF mRNA, the median, upper and lower quartile values were, respectively, 4,059, 9,245 and 1,707 for malignant tumours and 55,767, 79,002 and 41,410 for benign tissue. For basic FGF mRNA the median, upper and lower quartile values were, respectively, 4,267, 13,413 and zero for malignant tumours, and 39,915, 65,752 and 19,013 for benign tissue.

benign (Ke *et al.*, 1993) or malignant (Figure 1) breast cell lines. There was, however, a highly statistically significant difference between benign and malignant lesions in the mean level of expression of the mRNAs for either acidic FGF or basic FGF. Since cultured epithelial and myoepithelial-like cells derived from benign lesions fail to express detectable levels of acidic FGF mRNA (Barraclough *et al.*, 1990; Ke *et al.*, 1993), it is likely that much of this mRNA is derived from the stromal elements within the tissue samples. However, there was no consistent relationship between the levels of expression of the mRNAs for acidic FGF and basic FGF in each tumour, suggesting that these two mRNAs are independently expressed in the breast tumour samples.

Basic FGF and its mRNA are synthesised by rat (Barraclough *et al.*, 1990) and human (Ke *et al.*, 1993) myoepithelial-like cells in culture, but not by the epithelial cells within the mammary gland *in vivo*. Immunocytochemical studies have shown that basic FGF is localised primarily to the basement membrane and to a lesser extent to the myoepithelial cells in the normal rat and human breast and in benign breast tissue (Gomm *et al.*, 1991; Rudland *et al.*, 1993*a*); its cellular location in growing rat ductal structures is consistent with its synthesis by the myoepithelial-like but not the epithelial cells (Rudland *et al.*, 1993a). Myoepithelial cells are found in both normal and benign breast tissue, but are lost in human invasive breast carcinomas (Gusterson *et al.*, 1982; Rudland *et al.*, 1993*b*). The observation that invasive carcinomas generally express lower levels of basic FGF mRNA than does benign/normal tissue (this paper) and a similar previous report (Luqmani *et al.*, 1992) are possibly consistent with the loss of myoepithelial cells in the invasive carcinomas. Although in the present work there was no overall increase in the expression of the mRNA for basic FGF in the malignant breast tumours relative to that in the benign tissue, approximately 25% of the malignant tumours (excluding the '*in situ*' carcinomas, Table I) did express basic FGF mRNA at levels that were equivalent to, or higher than, those of the benign tissue. A similar proportion (20%) of the malignant human mammary cell lines tested expressed high level of basic FGF mRNA (Figure 1), and a similar proportion of cell lines expressing basic FGF mRNA has been previously reported (Li & Shipley, 1991; Luqmani *et al.*, 1992), although Luqmani *et al.* (1992) detected the mRNA for basic FGF in the cell line ZR-75-1 using an assay based on the polymerase chain reaction. All of these results together strongly suggest that, in some breast cancers, there is a population of tumour cells that acquire the ability to express high levels of basic FGF mRNA. Indeed, we have observed immunocytochemically detectable basic FGF in the malignant cells of some invasive human breast carcinomas using four independently isolated antibodies to basic FGF (Rudland *et al.*, 1993*b*), but the precise quantitation is dependent on the antibody used (unpublished results). Since basic FGF is angiogenic and angiogenesis is an important feature of tumour growth and metastasis (Weidner *et al.*, 1991), the ability of subclones of malignant epithelial cells to express basic FGF might favour the establishment of a more successfully metastasising breast cancer. However, since most of the released basic FGF is probably sequestered by nearby glycosaminoglycans *in vitro* (Fernig *et al.*, 1992) and *in vivo* (Rudland *et al.*, 1993*a*), the role of basic FGF in breast cancer is by no means clear cut.

We thank the North West Cancer Research Fund and the Cancer and Polio Research Fund for financial support. The help of Mrs A. Platt-Higgins with compiling data on the tumours, of Mr J. Carroll with the growth of the cultured cells and of Dr P. Manasse with the statistical analysis of the data is gratefully acknowledged.

### References

ALWINE, J.C., KEMP, D.J. & STARK, G. (1977). Method for the detection of specific RNAs in agarose gels by transfer to diazobenzyloxymethyl-paper and hybridization with DNA probes. *Proc. Natl Acad Sci. USA*, **12**, 5350–5354.

AVIV, H. & LEDER, P. (1972). Purification of biologically-active globin mRNA by chromatography on oligodeoxythymidylic acid–cellulose. *Proc. Natl Acad. Sci. USA*, **69**, 1408–1412.

BARRACLOUGH, R., KIMBELL, R. & RUDLAND, P.S. (1987). Differential control of mRNA levels for Thy-1 antigen and laminin in rat mammary epithelial and myoepithelial-like cells in culture. *J. Cell Physiol.*, **131**, 393–401.

BARRACLOUGH, R., FERNIG, D.G., RUDLAND, P.S. & SMITH, J.A. (1990). Synthesis of basic fibroblast growth factor upon differentiation of rat mammary epithelial to myoepithelial-like cells in culture. *J. Cell Physiol.*, **144**, 333–344.

CHIRGWIN, J.M., PRZYBYLA, A.E., MACDONALD, R.J. & RUTTER, W.J. (1979). Isolation of biologically active ribonucleic acid from sources enriched in ribonuclease. *Biochemistry*, **18**, 5294–5299.

ENGEL, L.W. & YOUNG, N.A. (1978). Human breast carcinoma cells in continuous culture: a review. *Cancer Res.*, **38**, 4327–4339.

FEINBERG, A.P. & VOGELSTEIN, B. (1984). A technique for radiolabelling DNA restriction endonuclease fragments to high specific activity. *Anal. Biochem.*, **137**, 266–267.

FERNIG, D.G., RUDLAND, P.S. & SMITH, J.A. (1992). Modulation of bFGF action by low affinity receptors in rat mammary myoepithelial-like cells in culture. *Growth Factors*, **7**, 27–39.

FOLKMAN, J. & SHING, Y. (1992). Angiogenesis. *J. Biol. Chem.*, **267**, 10931–10934.

GOMM, J.J., SMITH, J., RYALL, G.K., BAILLIE, R., TURNBULL, L. & COOMBES, R.C. (1991). Localisation of basic fibroblast growth factor and transforming growth factor beta 1 in the human mammary gland. *Cancer Res.*, **51**, 4685–4692.

GUSTERSON, B., WARBURTON, M.J., MITCHELL, D., ELLISON, M., NEVILLE, A.M. & RUDLAND, P.S. (1982). Distribution of myoepithelial cells and basement membrane proteins in normal breast and in benign and malignant breast diseases. *Cancer Res.*, **42**, 4763–4770.

HAN, J.H., STRATOWA, C. & RUTTER, W.J. (1987). Isolation of full-length putative rat lysophospholipase cDNA using improved methods for mRNA isolation and cDNA cloning. *Biochemistry*, **26**, 1617–1625.

KE, Y., FERNIG, D.G., WILKINSON, M.C., WINSTANLEY, J.H.R., SMITH, J.A., RUDLAND, P.S. & BARRACLOUGH, R. (1993). The expression of basic fibroblast growth factor and its receptor in cell lines derived from normal human mammary gland and a benign mammary lesion. *J. Cell Sci.*, **106**, 135–143.

LI, S. & SHIPLEY, G.D. (1991). Expression of multiple species of basic fibroblast growth factor mRNA and protein in normal and tumor-derived mammary epithelial cells in culture. *Cell Growth Different.*, **2**, 195–202.

LIDEREAU, R., CALLAHAN, R., DICKSON, C., PETERS, G., ESCOT, C. & ALI, I.U. (1988). Amplification of the int-2 gene in primary human breast tumours. *Oncogene Research*, **2**, 285–291.

LUQMANI, Y.A., GRAHAM, M. & COOMBES, R.C. (1992). Expression of basic fibroblast growth factor, FGFR1 and FGFR2 in normal and malignant human breast and comparison with other normal tissues. *Br. J. Cancer*, **66**, 273–280.

RUDLAND, P.S., PLATT-HIGGINS, A.M., WILKINSON, M.C. & FERNIG, D.G. (1993*a*). Immunocytochemical identification of basic fibroblast growth factor in the developing rat mammary gland: variations in location are dependent on glandular structure and differentiation. *J. Histochem. Cytochem.*, **41**, 887–898.

RUDLAND, P.S., LEINSTER, S.J., WINSTANLEY, J., GREEN, B., ATKINSON, M. & ZAKHOUR, H.D. (1993*b*). Immunocytochemical identification of cell types in benign and malignant breast diseases; variations in cell markers accompany the malignant state. *J. Histochem. Cytochem.*, **41**, 543–553.

SAMBROOK, J., FRITSCH, E.F. & MANIATIS, T. (1989). *Molecular Cloning: A Laboratory Manual.* Cold Spring Harbor Laboratory Press: Cold Spring Harbor, NY.

SLAMON, D.J., CLARK, G.M., WONG, S.G., LEVIN, W.J., ULLRICH, A. & MCGUIRE, W.L. (1987). Human breast cancer: correlation of relapse and survival with amplification of the HER-2/neu oncogene. *Science*, **235**, 177–182.

THEILLET, C., LE ROY, X., DE LAPEYRIERE, O., GROSGEORGES, J., ADNANE, J., RAYNAUD, S.D., SIMONY-LAFONTAINE, J., GOLDFARB, M., ESCOT, C., BIRNBAUM, D. & GAUDRAY, P. (1989). Amplification of FGF-related genes in human tumors: possible involvement of HST in breast carcinomas. *Oncogene*, **4**, 915–922.

WEIDNER, N., SEMPLE, J.P,. WELCH, W.R. & FOLKMAN, J. (1991). Tumor angiogenesis and metastasis – correlation in invasive breast carcinoma. *N. Engl. J. Med.*, **324**, 1–8.

WINSTANLEY, J., COOKE, T., MURRAY, G.D., PLATT-HIGGINS, S.A., GEORGE, W.D., HOLTS, S., MYSKOV, M., SPEDDING, A., BARRACLOUGH, B.R. & RUDLAND, P.S. (1991). The long term prognostic significance of c-erbB-2 in primary breast cancer. *Br. J. Cancer*, **63**, 447–450.